# Oncolytic Virotherapy: From Bench to Bedside

**DOI:** 10.3389/fcell.2021.790150

**Published:** 2021-11-26

**Authors:** Ludi Yang, Xiang Gu, Jie Yu, Shengfang Ge, Xianqun Fan

**Affiliations:** ^1^ Department of Ophthalmology, Ninth People’s Hospital, Shanghai JiaoTong University School of Medicine, Shanghai, China; ^2^ Shanghai Key Laboratory of Orbital Diseases and Ocular Oncology, Shanghai, China

**Keywords:** oncolytic virus, tumor, immunity, mechanisms, clinical applications

## Abstract

Oncolytic viruses are naturally occurring or genetically engineered viruses that can replicate preferentially in tumor cells and inhibit tumor growth. These viruses have been considered an effective anticancer strategy in recent years. They mainly function by direct oncolysis, inducing an anticancer immune response and expressing exogenous effector genes. Their multifunctional characteristics indicate good application prospects as cancer therapeutics, especially in combination with other therapies, such as radiotherapy, chemotherapy and immunotherapy. Therefore, it is necessary to comprehensively understand the utility of oncolytic viruses in cancer therapeutics. Here, we review the characteristics, antitumor mechanisms, clinical applications, deficiencies and associated solutions, and future prospects of oncolytic viruses.

## 1. Background

Cancer is the second leading cause of death worldwide with increased number of cases and cancer deaths. According to estimates by the WHO, in the next 20 years, the number of global cancer cases may increase by 60%, and cancer is a huge burden on society worldwide (Siegel et al., 2021). The effects of traditional treatment modalities remain limited. In recent years, several strategies such as immunotherapy and targeted therapy have been developed and have shown the potential to improve the outcome of numerous malignancies.

Oncolytic viruses are a novel type of multimechanistic therapeutic agent for cancer treatment and have the advantages of immunotherapy and targeted therapy (Kaufman et al., 2016). Modifying the genome of the oncolytic virus improves its tumor-targeting capability and oncolytic potential. Meanwhile, the host’s antitumor immunity can also be enhanced to achieve the purpose of treating cancer (Ramelyte et al., 2021). Oncolytic viruses are presently under evaluation in clinical and experimental trials, and some clinical research has demonstrated that oncolytic viruses significantly improve the prognosis of cancer patients and have a good safety profile (Lawler et al., 2017). In this review, we focus on the characteristics of oncolytic viruses and their mechanisms in tumor treatment. We discuss combinatorial strategies with other traditional treatments and immunotherapies and the challenges of oncolytic virotherapy.

## 2 Characteristics of Oncolytic Viruses

Viruses are particles that must parasitize living cells, causing infection and a related immune response (Boivin et al., 2002). The naked virion is composed of the core and the capsid (Lee et al., 2017; Miranda-Saksena et al., 2018). The core of the virus comprises one type of nucleic acid (DNA or RNA). This nucleic acid makes up the virus genome, storing all the genetic information of the virus and controlling its characteristics (Guerra et al., 2017). Enveloped viruses consist of a nucleocapsid wrapped in an envelope (Parvez, 2020). With the development of genetic engineering and virology techniques, it is now possible to modify the structure of viruses to make them useful for human therapy; as examples, inactivated viruses can be used for vaccines, viral vectors can be used for genetic engineering, and viruses can also be used for cell fusion in cell engineering (Khattar et al., 2011; Song and Viskovska, 2020).

Oncolytic viruses are a special type of virus that are naturally or genetically engineered and can replicate preferentially in tumor cells and inhibit tumor growth (Jennings et al., 2019; Harrington et al., 2019). According to the type of nucleic acid they contain, oncolytic viruses can be divided into DNA viruses and RNA viruses (Table 1) (Bommareddy et al., 2018a). The differences between DNA and RNA viruses are as follows. Compared with RNA viruses, the advantage of DNA viruses is that most DNA viruses can express high-fidelity polymerases, ensuring genetic integrity and efficient replication. (Kaufman et al., 2016). However, DNA viruses have many disadvantageous characteristics. First, the ability of DNA viruses to induce immunogenicity is not as good as that of RNA viruses. Reasons for this include inefficient viral DNA delivery levels and inadequate DNA-sensing machinery and nucleic acid-sensing pattern recognition receptor expression levels (McNamara et al., 20152015). Second, there is also a risk that viral DNA will integrate into the host genome, inactivating tumor suppressors or activating oncogenes (Liu, 2019). Third, most RNA oncolytic viruses have more efficient delivery processes and are more advantageous for specifically killing central nervous system tumors. RNA oncolytic viruses are much smaller than DNA viruses, and they spread more easily throughout the human body, they can even cross the blood-brain barrier (Yu et al., 2011).

**TABLE 1:** The unique characteristics of oncolytic virus.

	DNA				RNA	Rhabdovirus	Picornavirus	Togavirus	Reovirus
	Parvovirus	Adenovirus	Herpesvirus	Poxvirus	Paramyxovirus				
	ssDNA	dsDNA	dsDNA	dsDNA	ss (−)RNA	ss (−)RNA	ss (+)RNA	ss (+)RNA	dsRNA
Genome size	5–6 kb	26–45 kb	120–240 kb	130–280 kb	15.2–15.9 kb	11–15 kb	7–8 kb	9.7–11.8 kb	10 segments of double-stranded (ds) RNA
Virion	Naked	Naked	Enveloped	Enveloped	Enveloped	Enveloped	Naked	Enveloped	Naked
Capsid symmetry	Icosahedral	Icosahedral	Icosahedral	Complex	Helical	Helical	Icosahedral	Icosahedral	Icosahedral
VAP	NA	spike or fiber associated with each penton base of the capsid	gp350/gp220	A27L, M115L, A26	HA	gpG	VP1-VP3	E1, E2	σ1s
Cell receptor	Sialic acid residues	CAR, CD46, VCAM1	HVEM, nectin 1, nectin 2	GAGs, EFC	SLAM, CD46, Neuraminidase	LDLR	CD155, CAR, ICAM-1, DAF	phospholipid receptors	JAM-A
					Receptor, sialoglycoconjugates				
type of penetration	Viropexis	Viropexis	Viropexis	Fusion	Fusion	Viropexis	Viropexis	Viropexis	NA
Ability to penetrate BBB	+	−	−	−	−/+	−	−/+	+	+
transgene capacity	+	++	+++	+++	+	+	+	NA	+++
Immunogenicity	+	−	−	−	−	−	+/−	+	−
Antivirals	−	+	+	+	−	−	−	NA	−

NA, not applicable.

## 3 Mechanisms of Oncolytic Viruses

### 3.1 Selectivity

Tumor cells are different from normal cells in terms of genetics and physiology. As a result of the abnormal antiviral ability and signaling pathways of tumor cells and tumor-specific promoters or mRNAs, oncolytic viruses can specifically target tumor cells without replicating and growing in healthy cells. In addition, the differential expression of apoptosis-related genes or surface receptor proteins between tumor and normal cells is also important for virus targeting. The selectivity of viruses is an inherent guarantee of their safety in clinical applications.

Antiviral signaling pathways (such as the IFN pathway) in normal cells can eliminate viruses. In tumor cells, there are mutations or deletions among the key protein-coding genes of these signaling pathways. Therefore, oncolytic viruses can use dysregulated signaling pathways in tumor cells to promote virus replication, infection and spread (Stewart et al., 2011; Ilkow et al., 2014; Kurokawa et al., 2018). The matrix (M) protein is commonly present in negative stranded RNA viruses, lining the inner side of the envelope of the virions. The M protein of Vesicular stomatitis virus (VSV) is a structural protein and virulence factor that plays a significant role in virus assembly and suppression of host gene expression. M protein variants retain virus assembly functions but can not inhibit host IFN gene expression. VSVs expressing M protein variants cannot replicate in normal tissue cells due to the activation of the IFN signaling pathway. However, they can selectively replicate in tumor cells with defective IFN immune responses (Stewart et al., 2011). In addition, there are other abnormal nonimmune signaling pathways in tumor cells. For example, ICP4 is a key protein required for HSV replication that can be activated by ELK, a downstream protein of RAS. A mutant HSVwhose expression of ICP4 is controlled by activation of ELK has the selective ability because the RAS signaling pathway is generally silent in normal cells, while it is activated in tumor cells (Pan et al., 2009). Some wild-type viruses, such as reovirus, have shown a good natural preference for tumor cells with overactive RAS, and these viruses can function without engineering (Shmulevitz et al., 2005; Carew et al., 2017).

Some specific promoters can regulate the transcription and expression of key viral replication genes. Controlling key viral expression genes with such promoters can restrict the virus from being highly expressed in tumor tissues (Deweese et al., 2001; Cheng et al., 2015; Zhang et al., 2015). E2F-1 (Tsukuda et al., 2002), hTERT (Sato et al., 2013) and HIF-1 (Longo et al., 2011) are promoters with high expression in tumor tissues and low expression in normal tissues. Placing a key viral replication gene (such as E1A and E1B) under these specific promoters stimulates the expression of the key gene in tumor tissues, which can enhance the selectivity of the virus (Yoon et al., 2001).

Inserting tissue-specific miRNA sequences into the oncolytic virus genome can also regulate the expression of key genes, preventing the replication of viruses in normal tissue cells (Yao et al., 2014). miR199 is a miRNA that is downregulated in hepatocellular carcinoma (HCC) compared to in the normal liver (Lou et al., 2018). Adenovirus-199T (Ad-199T) is a recombinant adenovirus in which copies of DNA segments complementary to miR199 are inserted within the essential adenovirus replication gene E1A. Ad-199T can replicate in cells lacking miR-199 to make virus replication and cytolytic effects specifically selective for HCC cells (Callegari et al., 2013).

Viral selectivity can also be achieved by targeting key genes that play an important role in viral replication in normal cells. The genes themselves have to be unrelated to the replication of the virus in the tumor. The E1B-55 K protein, which prevents premature apoptosis, is expressed among wild-type adenoviruses (Dix et al., 2000). The p53 gene is an important apoptosis-inducing gene in normal cells. Inactivation of the p53 gene is an important feature of tumor cells. Viral replication depends on the inhibition of host cell apoptosis, which is related to the function of p53. An oncolytic adenovirus with E1B gene deletion cannot replicate in normal cells due to the dysfunction of apoptosis inhibition, while in p53-deficient tumor cells, replication is not affected (Bischoff et al., 1996).

Another way to make a virus tumor-specific is genetically modifying the viral capsid protein to achieve tumor cell-specific binding. For example, uPAR is a highly expressed receptor on the surface of a variety of tumor cells. uPAR is closely related to tumor aggressiveness and angiogenesis. The MV-h-uPA construct contains an added fragment that binds to uPAR and can specifically infect tumor cells with high expression of uPAR through the receptor-ligand pathway (Jing et al., 2014). Furthermore, inserting lysine residues into ciliated proteins allows the virus to bind to tumor cells that widely express heparin sulfate receptors. HER-2, a surface molecule, is hyperexpressed in 15–20% mammary and ovary carcinomas. HSVs can be engineered to target HER2 by replacing the Ig-folded core in the receptor-binding virion glycoprotein D with an anti-HER-2 single-chain antibody (Menotti et al., 2009). HSV also mediates highly efficient infection in glioblastoma cells in which EGFR is highly expressed, by combining engineered viral glycoproteins, gD and gB (Uchida et al., 2013).

### 3.2 Tumor Lysis

**FIGURE 1:**
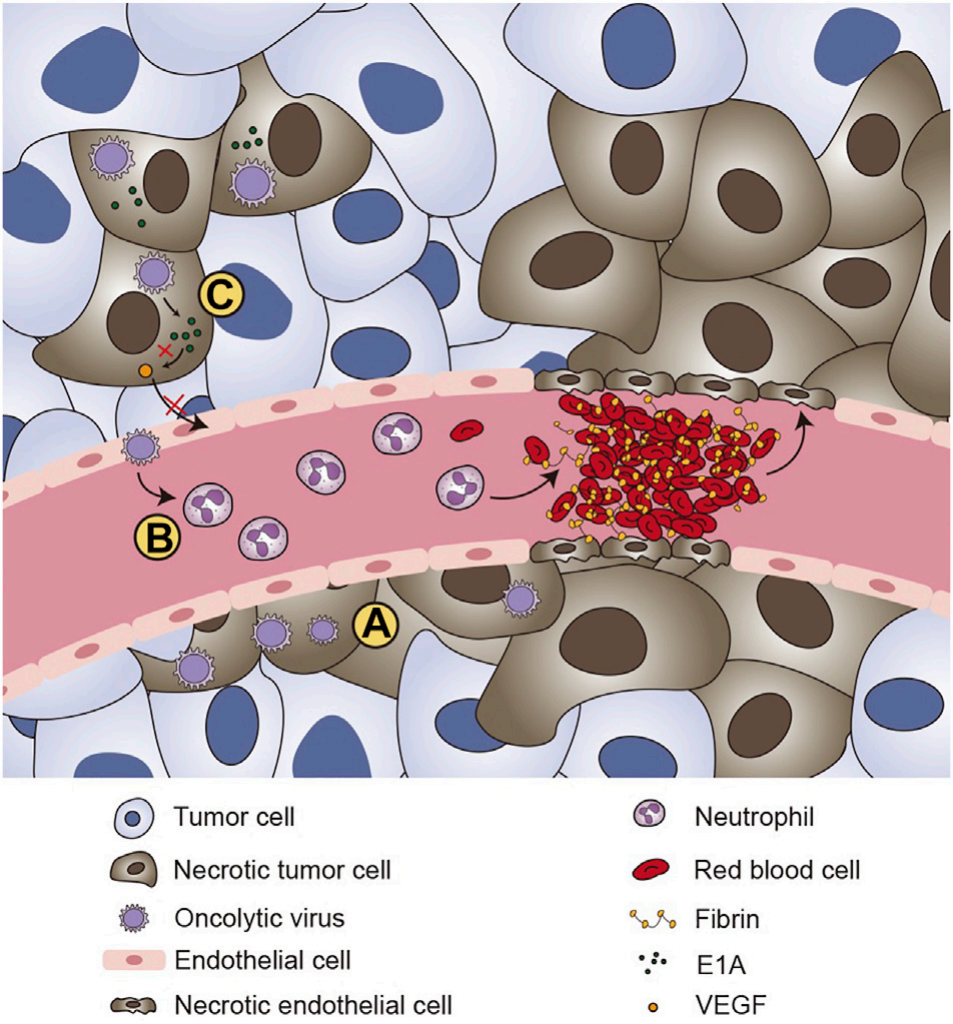
The effects of tumor lysis of oncolytic virus. **(A)** Oncolytic viruses are able to kill tumor cells directly. **(B)** After infecting the endothelium of the tumor vasculature, oncolytic viruses are able to recruit neutrophil cells and mediate the formation of clots and vascular collapse, which induce the ischemic death of tumor cells. **(C)** VEGF plays an important role in the regulation of angiogenesis and tumor growth. Some specific oncolytic viruses, such as adenovirus, can express the E1A protein, which can downregulate VEGF by interacting with angiogenic proteins, thereby affecting new blood vessels in the tumor microenvironment and ultimately achieving oncolytic effects.

The effects of tumor lysis can be categorized into direct effects on cells and effects on tumor vasculature (Figure 1).

Oncolytic viruses use tumor cells as a processing factory and replicate in large quantities. During proliferation, the oncolytic virus can block the synthesis of cellular nucleic acids and proteins, which causes cell metabolism dysfunction and ultimately lyses tumor cells. In addition, the nucleus, lysosomes, endoplasmic reticulum and mitochondria of the infected cells can all be damaged as a result of high viral replication volumes. In addition to the effects of replication, the structures of oncolytic viruses, such as the capsid protein, can also cause direct oncolysis.

Tumor neovascularization is a key mechanism by which tumors maintain their growth and development (Lugano et al., 2020). Inhibiting tumor neovascularization can cut off the supply of tumor oxygen and nutrients, thereby inhibiting tumor growth and metastasis (Qian and Pezzella, 2018). Oncolytic viruses have potent antiangiogenic properties and can induce vascular collapse, which in turn causes tumor cell death (Gholami et al., 2016). HSV is able to infect the endothelium of the tumor vasculature by recruiting inflammatory cells that lead to the formation of local microthrombi (Breitbach et al., 2007). This phenomenon has been verified in various cancers, such as ovarian cancer, glioma, and rhabdomyosarcoma. Breitbach C. et al. revealed that vaccinia viruses (VVs) can replicate within endothelial cells of the tumor-associated vasculature (Breitbach et al., 2013). Over time, the tumor’s blood vessel density gradually decreases, and neutrophils continue to accumulate, resulting in decreased tumor cell perfusion and the ischemic death of tumor cells. Vascular endothelial growth factor (VEGF) is a highly specific vascular endothelial cell growth factor. It plays critical roles in the migration, proliferation and angiogenesis of vascular endothelial cells, which are closely related to tumor progression. Adenovirus can express E1A protein, which can downregulate VEGF by interacting with angiogenic cell proteins, thereby affecting new blood vessels formation in the tumor microenvironment and ultimately achieving oncolytic effects (Ye et al., 2006).

### 3.3 Antitumor Immunity

#### 3.3.1 Innate Immunity

Tumor cells and their microenvironment usually express cytokines (such as IL-10 and TGFβ) with immunosuppressive functions, which inactivate effector immune cells and recruit immunosuppressive cells to the tumor (Borsig, 2018). In some cases, there are few immune cells in the tumor microenvironment, which is known as a “cold” microenvironment. Oncolytic viruses are capable of reversing this immunosuppressive microenvironment, turning “cold” tumors into “hot” tumors by changing the cytokine environment and promoting immune cell maturation (Galon and Bruni, 2019).

Following viral infection, oncolytic viruses can induce the death of tumor cells by mediating immunogenic cells. Viral elements (tumor-associated antigens (TAAs) and viral pathogen-associated molecular patterns (PAMPs)) and cell-derived damage-associated molecular patterns (DAMPs) can be released (Yoo et al., 2013). Pattern recognition receptors (PRRs) such as the family of Toll-like receptors can recognize TAAs, PAMPs and DAMPs and trigger MYD88-dependent and TRIF-dependent signaling (Chen et al., 2018). In addition, there are other sensing elements, such as RIG-I-like receptors (RLRs: RIG-1 and MDA5), PKR and cyclic GMP–AMP synthase (cGAS)–stimulator of interferon genes (STING). PKR, RIG-I and MDA5 recognize RNA, while the cytosolic sensor cGAS detects DNA. RIG-I and MDA-5 are thought to recognize ssRNA and dsRNA, stimulating the release of NF-κB, IRF3, and IRF7(Lin et al., 2019). In the process of viral infection and invasion, cytoplasmic DNA is recognized as a danger signal by the DNA sensor cGAS, and the downstream protein STING is used as an adaptor molecule to recognize cGAMP and activate downstream signals to promote the production of type I interferons and other cytokines (Kato et al., 2017).

These cytokines play a critical role in the recruitment and activation of innate immune cells, such as dendritic cells (DCs) and natural killer (NK) cells, which can reverse the immunosuppressive microenvironment in tumors. For example, dendritic cells loaded with human melanoma Mel888 cells (DC-Mel) cannot normally respond to PRR-PAMP signaling, which leads to low antitumor immunity. Reovirus can reverse the dysfunction of DCs-Mel by upregulating the secretion of costimulatory factors and cytokines. Reovirus can reduce the secretion of IL-10 from immunosuppressive cells and induce the production of proinflammatory factors such as the macrophage inflammatory protein MIP-1α/β. These cytokines can activate DCs and mediate a variety of immune responses (Prestwich et al., 2009).

### 3.3.2 Adaptive Immunity

**FIGURE 2:**
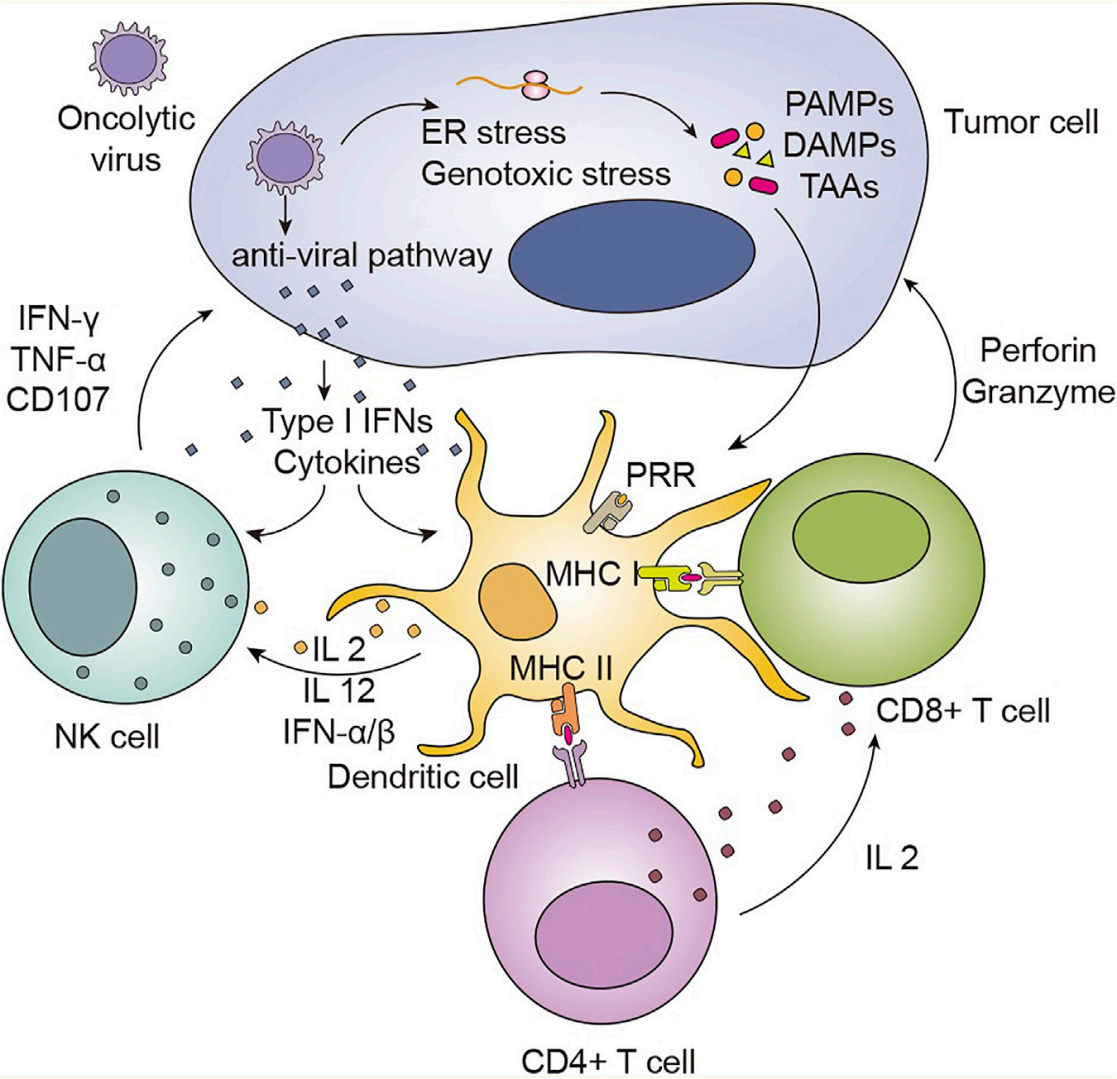
Oncolytic viruses can induce host systemic anti-tumor immunity to kill tumor cells. Following viral infection, viral replication leads to ER stress and genotoxic stress in tumor cells, which results in the release of the viral elements TAAs, PAMPs, and DAMPs. Sensing elements, such as PRRs, can recognize these molecules, which leads to immune cell activation and inflammatory signal transduction. With virus replication, antiviral pathways can be activated. This activation induces the production of cytokines and type I IFNs, which mediate the activation and maturation of immune cells, such as DCs and NK cells. NK cells migrate to the tumor area under the action of chemokines, such as IL-12, IL-2, and IFN-α/β, and exert antitumoral properties by releasing IFN-γ, TNF-α and CD107. Mature DCs can present antigenic peptides within the context of MHC I molecules to CD4^+^ T cells and within the context of MHC II molecules to CD8^+^ T cells. CD8^+^ T cells are also activated through the stimulation of CD4^+^ T cells. The activation of CD4^+^ T cells by DCs helps to activate CD8^+^ T cells by inducing the production of cytokines such as IL‐2. The recognition of tumor surface antigens by antibodies can trigger CTL killing of tumor cells through Fas-FasL interactions, TNF‐TNFR signaling, and the perforin/granzyme pathway.

Most tumor cells do not express MHC molecules or express them at a low level, resulting in tumor cells that lack or weakly present tumor antigens. Such tumor cells do not induce cytotoxic T lymphocyte (CTL) activation; thus, no immune-system-based tumor-cell killing occurs (Axelrod et al., 2019). Oncolytic viruses are capable of promoting DC maturation and increasing their antigen presentation ability (Raja et al., 2018). Mature DCs (mDCs) can present antigenic peptides on MHC I and MHC II molecules to CD8^+^ and CD4^+^ T cells, respectively, and deliver costimulatory signals that activate T cells (Pardee et al., 2015). In a murine ovarian cancer model, J. Patrick Murphy et al. revealed that tumor MHC-1 ligand expression was increased after treatment with oncolytic reovirus (Murphy et al., 2019). An increase in MHC was detected not only in tumor cells but also in spleen tissues. The same results were also observed in CMT64 lung adenocarcinoma cells infected with oncolytic adenovirus. In a model in which DCs are incubated with tumor cells, tumor cells not infected with reoviruses cannot induce the killing response of cytotoxic T cells, but this response can be induced by infected tumor cells (Figure 2).

As a new potential cancer therapy, oncolytic viruses can prevent tumor recurrence by establishing immune memory in the body. Minjun Yu et al. induced lymphoma regression in mice by administering Sindbis virus vectors in combination with α4-1BB monoclonal antibodies (Yu et al., 2019). In addition, all mice treated with Sindbis virus vectors and α4-1BB monoclonal antibodies were proven to have durable antitumor immune memory. Havunen et al. constructed an oncolytic adenovirus with insertion of the human IL-2 gene and the human tumor necrosis factor α (hTNF-α) gene (Havunen et al., 2017). They combined the transformed adenovirus with tumor-infiltrating lymphocytes (TILs) to form the Ad5/3-E2F-d24-hTNFα-IRES-hIL2 (TILT-123) strategy, which was tested in Syrian hamsters with tumors formed from transplanted pancreatic cancer cells. The results indicated that oncolytic viruses carrying the IL-2 and TNF-α genes could increase the frequency of CD4^+^ and CD8^+^ TILs and strengthen the persistence and proliferation of T cells. After treatment, Syrian hamsters were again inoculated with the same pancreatic cancer cells, and the tumor recurrence rate was lower than that of Syrian hamsters that received oncolytic virus treatment alone.

### 3.4 Transgene Expression

Oncolytic viruses can also be genetically modified to serve as vectors to express therapeutic genes to kill tumor cells in multiple ways, effectively avoiding the current common drug resistance problem of single-target anticancer drugs (Marino et al., 2017). This strategy is known as “arming” and is similar to combining an oncolytic virus with a cancer-killing “accomplice".

#### 3.4.1 Suicide Genes

Suicide gene therapy is known as virus-directed enzymatic prodrug therapy (VDEPT) (Zhang et al., 2010; Karjoo et al., 2016). Suicide gene therapy involves the introduction of a gene encoding a specific sensitivity factor into tumor cells, which causes the cells to be specifically sensitive to a specific originally nontoxic or low-toxicity drug, thereby causing tumor cell death. This expressed sensitivity gene is called a suicide gene or a drug sensitivity gene. T601 is a VV with TK and RR knocked out that expresses the fusion suicide gene FCU1(Foloppe et al., 2019). FCU1 is a product of the fusion of the yeast cytosine deaminase gene and the uracil phosphoribosyltransferase gene (Husseini et al., 2017). Yeast cytosine deaminase can convert the prodrug 5-FC into the chemotherapeutic drug 5-fluorouracil (5-FU), while uracil phosphoribosyltransferase can further convert 5-FU into 5-fluorouracil triphosphate (FUTP) and 5-fluorodeoxyuracil monophosphate (FdUMP). FUTP inhibits the activity of RNA, and FdUMP inhibits the synthesis of DNA and can reduce the degradation of 5-FU, thereby killing tumor cells (Dias et al., 2010).

#### 3.4.2 Anti-angiogenesis Genes

Angiogenesis is essential for tumor progression. It has been proven that angiogenesis inhibitors have antitumor effects. Endostatin and angiostatin are two broad-spectrum angiogenesis inhibitors (Gonzalez et al., 2018). However, endostatin and angiostatin have no direct tumor cell killing effect and must be continuously transported to the tumor microenvironment. Due to their short half-life in serum, low solubility and poor stability, angiostatin and endostatin cannot function in a traditional manner (Marshall, 2002). Oncolytic viruses genetically modified to continuously express angiogenesis inhibitors in the tumor microenvironment can inhibit angiogenesis factors and significantly reduce the activation and migration of endothelial cells (Indraccolo et al., 2002). Hutzen B et al. constructed the measles virus (MV) variants MV-hE: A and MV-Me. These viruses can express human and mouse endostatin/angiostatin fusion proteins, respectively. In medulloblastoma, the target gene is continually expressed as the oncolytic virus replicates, which leads to the inhibition of angiogenesis factors and induces the death of tumor cells. (Hutzen et al., 2014).

#### 3.4.3 Immune-Related Genes

Inserting cytokine-related genes into the oncolytic virus genome can effectively increase the local immune inflammatory mediators in the tumor microenvironment, thereby activating the body’s antitumor immune response and enhancing the antitumor effect of the oncolytic virus. To date, various cytokine genes have been used to generate recombinant oncolytic viruses, such as interleukin, colony-stimulating factor, tumor necrosis factor, and chemokine genes (Zhu et al., 2012; Conrad et al., 2015; Hirvinen et al., 2015; Bourgeois-Daigneault et al., 2016; Deng et al., 2016). GM-CSF is currently the most widely used cytokine in clinical practice (Chon et al., 2019). It has been integrated into the genomes of various oncolytic viruses and has shown a certain degree of therapeutic efficacy in preclinical studies (Liu et al., 2013). The first oncolytic virus approved by the FDA, T-VEC, is a version of HSV-1 with GM-CSF inserted into its genome (Andtbacka et al., 2015).

In addition, viruses can be engineered to express other immune factor genes to enhance the body’s antitumor immune response. The oncolytic virus G47Δ-IL-12, derived from an HSV-1 strain, has a strong oncolytic effect on malignant gliomas (Saha et al., 2017). This virus can significantly promote the killing of tumor cells and virus-infected cells by expressing IL-12. A preclinical model showed that monotherapy with IL12-expressing HSV is sufficient to eradicate glioma and provides resistance to rechallenge with high-grade gliomas, even without immune checkpoint blockade (Alessandrini et al., 2019). Ad-ZD55 is an adenovirus derived from the deletion of the E1B-55 kD gene (Liu and Gu, 2006). Ad-ZD55-TRAIL and Ad-ZD55-IL-24 are viruses derived from Ad-ZD55 with the TRAIL gene and IL-24 gene inserted, respectively (Zhao et al., 2006). TRAIL and IL-24 can interact and promote each other, further inducing substantial tumor apoptosis by activating intracellular tumor-killing mechanisms. Oncolytic adenoviruses can express the chemokines MIP-1α or RANTES, which induce the recruitment of DCs and enhance the antitumor immune response (Edukulla et al., 2009).

#### 3.4.4 Tumor Microenvironment Related Genes

The formation of the tumor microenvironment may prevent oncolytic viruses from exerting their effects. To eliminate the unfavorable factors caused by the tumor microenvironment, viruses can be genetically modified or altered by other methods to enhance their efficacy. The extracellular matrix (ECM) of solid tumors can affect the infection and spread of therapeutic viruses (Mok et al., 2007). Matrix-degrading enzymes improve tumor permeability by degrading ECM, increasing the spread of viruses and the concentration of viruses in tumor cells. Li et al. constructed an oncolytic adenovirus expressing decorin (DCN) (Li et al., 2018). The results of their experiments showed that the virus mediated the expression of DCN, which downregulated the main components of the ECM (such as collagens I and III), in pancreatic cancer tumor tissues. This effect resulted in the accelerated spread of the virus in mice with pancreatic tumors *in situ* and induced tumor cell apoptosis.

## 4. Oncolytic Viruses in Cancer

Given the antitumor properties of oncolytic viruses, a variety of oncolytic viruses with tumor-killing effects have been developed. The clinical applications of oncolytic viruses are introduced below according to their types (TABLE 2).

**TABLE 2:** Oncolytic virus anti-tumor clinical trials.

Virus	Modification	Virus administration	Status	Indication	Therapeutic Approach	Clinical trials	Status
*Adenovirus*							
DNX-2401	Δ24-RGD insertion	Intratumoural	I	Glioblastoma, ovarian cancer	Combination or OV only	NCT03178032 NCT01956734	Active
							Completed
VCN-01	PH20 hyaluronidase	Intratumoural	I	Pancreatic cancer, Retinoblastoma, Head and Neck Neoplasms	Combination or OV only	NCT03799744 NCT03284268	Recruiting
				Osteosarcoma			Recruiting
CG0070	GM-CSF and E3 deletion	Intratumoural	I-III	Bladder Cancer	Combination or OV only	NCT04610671 NCT02365818	Recruiting
							Completed
Colo-Ad1	Chimeric Ad11/3 group B	Intratumoural	I	Colon cancer, NSCLC, Renal cell carcinoma, Bladder cancer	OV only	NCT02053220	Completed
ICOVIR5	Modified DNX-2401-E2F promoter opimitized	Intravenous	I-II	Melanoma, Solid Tumors	OV only	NCT01864759	Completed
						NCT01844661	Completed
Ad5-yCD/mutTKSR39rephIL12	Ad serotype 5; insertion of IL12, yeast cytosine deaminase and TKSR39	Intratumoural	I	Prostate Cancer, Pancreatic Cancer	OV only	NCT02555397	Recruiting
						NCT03281382	Recruiting
H101	E1B deletion, partial E3 deletion	Intratumoural	I-III	Hepatocellular Carcinoma and head and neck cancer	Combination	NCT03790059 NCT03780049	Recruiting
							Recruiting
ProstAtak	TK insertion	Intratumoural	II-III	Prostate Cancer	Combination or OV only	NCT01436968 NCT02768363	Recruiting
							Active
*Herpesvirus*							
T-VEC	ICP34.5 deletion, US11 deletion, GM-CSF insertion	Intratumoural	I-III	Melanoma, head and neck cancer and pancreatic cancer	Combination or OV only	NCT03086642	Recruiting
						NCT03069378	Recruiting
						NCT01368276	Completed
HF10	UL56 deletion, selected for single partial copy of UL52	Intratumoural	I-II	Breast cancer, melanoma, and pancreatic cancer	Combination or OV only	NCT03153085 NCT03259425	Completed
							Terminated
HSV1716	ICP34.5 deletion	Intratumoural	I-II	Malignant Pleural Mesothelioma, Rhabdomyosarcoma, Osteosarcoma, Anaplastic Oligodendroglioma	Combination or OV only	NCT01721018 NCT00931931 NCT02031965	Completed
							Completed
							Terminated
G207	ICP34.5 deletion	Intratumoural	I-II	Glioblastoma	OV only	NCT03911388	Recruiting
	UL39 disruption					NCT04482933	Not yet recruiting
*Measles Virus*							
MV-NIS	NIS insertion	intraperitoneal and Intratumoural	I-II	Myeloma, Ovarian cancer, Mesothelioma, NSCLC	Combination or OV only	NCT02919449 NCT02364713	Terminated
							Recruiting
MV-CEA	CEA insertion	Intraperitoneal, Intratumoural and Intravenous	I-II	Glioblastoma, Ovarian Cancer, Fallopian Tube Endometrioid Adenocarcinoma	OV only	NCT00390299 NCT00408590 NCT02068794	Completed
							Completed
							Recruiting
*Vaccinia Virus*							
Pexa-vac (JX-594)	GM-CSF insertion, TK disruption	Intratumoural and Intravenous	I-III	Melanoma, liver cancer, colorectal cancer, breast cancer, and hepatocellular carcinoma	Combination or OV only	NCT02562755 NCT00554372 NCT00629759	Completed
							Completed
							Completed
GL-ONC1	TK disruption, haemagglutin disruption, F14.5 L disruption	Intraperitoneal, Intratumoural and Intravenous	I-II	Lung cancer, head and neck cancer, and mesothelioma	Combination or OV only	NCT02759588 NCT01443260	Active
							Completed
*Reovirus*							
Reolysin	None	Intravenous and intratumoral	I-III	Glioma, sarcomas, colorectal cancer, NSCLC, ovarian cancer, melanoma, pancreatic cancer, multiple myeloma, head and neck cancer	Combination or OV only	NCT01166542 NCT02620423 NCT01656538	Completed
							Completed
							Completed
*Newcastle Disease Virus*							
PV701	None	Intravenous	I	Squamous Cell Carcinoma of the Larynx, Salivary Gland Cancer	OV only	NCT00081211	Terminated
						NCT00055705	Completed
NDV-HUJ	None	Intravenous	I-II	Glioblastoma, Sarcoma, Neuroblastoma	OV only	NCT01174537	Withdrawn
*Coxsackievirus*							
Cavatak (CVA21)	None	Intravenous and Intratumoural	I-II	Bladder Cancer, NSCLC, Uveal Melanoma, Breast Cancer, Prostate Cancer, Head and Neck Cancer	Combination or OV only	NCT00636558 NCT03408587 NCT01636882	Completed
							Completed
							Complete
*Vesicular Stomatitis Virus*							
VSV-hIFNβ	IFN-β insertion	Intratumoural	I	Head and Neck Squamous Cell Carcinoma, NSCLC, Hepatocellular Carcinoma	Combination	NCT02923466 NCT01628640	Active
							Active

### 4.1 Different Types of Viruses

#### 4.1.1 Adenoviruses

Adenoviruses are non-enveloped viruses with double-stranded DNA genomes (Greber and Flatt, 2019). They have a large genome (approximately 35 kb), which is convenient for genetic modification (Shen et al., 2009). Human adenovirus (HAdV) can be divided into more than 50 serotypes, which can be classified into seven species (HAdV-A to -G) (Marrugal-Lorenzo et al., 2019). CG0070 and ONYX-015 are two classic examples of oncolytic adenoviruses. CG0070 has antitumor activity by expressing the immunoregulatory factor GM-CSF. Nagarajan Ramesh et al. investigated antitumor efficacy of CG0070 in several bladder transitional cell carcinoma models, which has shown good tumor specificity and oncolytic effects (Ramesh et al., 2006). Barker et al. used ONYX-015, which has a deletion of the E1B gene, in the therapy of head and neck cancer, pancreatic cancer and recurrent granulation tumors, The objective remission rate of patients in clinical trials has been improved (Barker and Berk, 1987). Studies of Ilya V Ulasov et al. showed that CRAd-S-pk7 cells selectively target and penetrate tumor metastases, effectively delivering the CRAd-S-pk7 virus (Ulasov et al., 2007). Ilya V Ulasov established the effectiveness and safety of NSC-CRAd-S-pk7 in patients with high-grade glioma in a phase I clinical trial (Fares et al., 2021). Adenoviruses are also applied in celyvir, an advanced treatment that combines mesenchymal stem cells (MSCs) loaded with ICOVIR-5, an oncolytic adenovirus. David Ruano et al. demonstrated that MSCs ensure sufficient viral load by providing an immune shield to the virus *in vitro* (Ruano et al., 2020).

#### 4.1.2 Herpesviruses

Herpesviruses are enveloped, icosahedral viruses containing double-stranded DNA, approximately 120–240 kb. A total of more than 100 Herpesviruses have been found, which can be divided into three categories: α, β, and γ(Heldwein, 2018). Herpesviruses have demonstrated oncolytic properties in a variety of tumor types. T-VEC is an oncolytic virus derived from HSV-1 encoding GM-CSF. Robert H I Andtbacka et al. conducted a phase III clinical trial, which demonstrated that T-VEC has good and durable therapeutic effects in the treatment of advanced melanoma (Andtbacka et al., 2015). In 2015, T-VEC was approved by the United States FDA for the treatment of metastatic melanoma (Poh, 2016). T Mineta et al. created G207, a double mutant of the herpes simplex virus (HSV) type with deletion of ICP34.5 and insertional inactivation of UL39 (Mineta et al., 1995). Radiographic and neuropathological examinations have shown that G207 has antitumor activity and can be safely administered multiple times for the treatment of glioma (Markert et al., 2009). HF10 is a spontaneously mutated oncolytic virus derived from HSV-1. Nakao et al. treated six breast cancer patients with HF10 (Nakao et al., 2007). Two superficial nodules were selected in all patients with metastatic breast cancer, one of which was injected with the virus directly and one of which served as a control. After treatment, none of the patients had obvious adverse reactions, and tumor cell death in the nodules was observed by histopathological methods.

#### 4.1.3 Measles Virus

Measles virus is spherical or filamentous, with a diameter of about 120–250 nm. The core inside is a single negative strand RNA, without segmentation, and the full length of the genome is about 16 kb. Many studies have confirmed that MV has killing activity in prostate cancer, mesothelioma, ovarian cancer and other tumor cell lines (Hasegawa et al., 2006; Msaouel et al., 2009; Xia et al., 2019; Delaunay et al., 2020). Loi K Phuong et al. constructed MV-CEA, an MV that carries a gene encoding a carcinoembryonic antigen (Phuong et al., 2003). In a clinical trial, 21 patients with relapsed ovarian cancer received multiple intraperitoneal injections of MV-CEA within 4 weeks: 14 of them had stable disease (SD) after treatment, and five patients had decreased CA125 levels, which indicated that MV-CEA has good tumor-killing activity. Therefore, MV-CEA is a potentially effective antitumor drug.

#### 4.1.4 Vaccinia Virus

Vaccinia virus is an enveloped virus. The genome of the vaccinia virus is made up of double stranded DNA of nearly 200 kb and replicates in the cytoplasm of the host cell (Greseth et al., 2012). Several genetically modified VV strains have been extensively tested in clinical trials (Guo et al., 2019). J H Kim et al. developed JX-594, a targeted, thymidine kinase (−) vaccinia virus expressing human GM-CSF. It can replicate in cancer cells and activate the body’s antitumor immune response by expressing GM-CSF(Kim et al., 2006; Heo et al., 2013). A phase 1b study showed that administration of JX-594 via intravenous infusion once every 2 weeks was safe and well tolerated (Park et al., 2015). A new phase 1/2 study in early-stage tumors revealed that the combination of JX-594 and chemotherapy had a synergistic effect. However, the phase III study of JX-594/Sorafenib was terminated early. Combination therapy beyond Sorafenib monotherapy has yet failed to demonstrate superiority. Though it’s a clinical failure for oncolytic virotherapy, Sorafenib was further shown to inhibit vaccinia virus replication in tumor cells, which indicates that JX-594 may not very suitable in the combination with Sorafenib (Gilchrist et al., 2020).

#### 4.1.5 Reoviruses

Reoviruses are naked viruses with 10 segments of double-stranded DNA genomes. They are widely present in human respiratory and digestive tracts (Müller et al., 2020). Pelareorep, a serotype 3 reovirus, showed encouraging antitumor efficacy in combination with pembrolizumab in patients with advanced pancreatic adenocarcinoma (Mahalingam et al., 2020). Studies have shown that intratumoral injection and intravenous applications of reovirus are safe and tolerable (Samson et al., 2018). In clinical studies of intravenous injection of reovirus combined with administration of chemotherapeutics, such as carboplatin and paclitaxel, the patient survival period was significantly prolonged, and tumor progression has been inhibited (Kyula et al., 2012).

#### 4.1.6 Newcastle Disease Virus

Newcastle disease virus is a ssRNA virus with an envelope. Virus particles are pleomorphic, including round, elliptical, and long rod-shaped. Since the Newcastle disease virus (NDV) was first discovered to replicate and kill tumor cells in 1955, researchers have investigated its potential as a tumor treatment (Hanson and Brandly, 1955; Wei et al., 2015). Due to tumor-specific defects in the IFN-mediated antiviral response, PV701, a natural attenuated virus, kills tumors selectively (Pecora et al., 2002). Seventy-nine patients with solid tumors were enrolled in a phase I clinical trial evaluating intravenously injected of PV701(Lorence et al., 2003). Among them, one patient with tonsil squamous cell carcinoma experienced tumor regression after treatment, and seven patients with different tumor types experienced tumor shrinkage. However, a patient with lung metastases died. The autopsy revealed severe inflammation in the lung tissue, which might have been related to the PV701 treatment.

#### 4.1.7 Coxsackieviruses

Coxsackieviruses are non-enveloped viruses with single-stranded and positive polarity RNA genome and belong to the picornavirus family of the enterovirus genus (Kaese et al., 2017). Coxsackieviruses can be divided into two types, A and B. They are common viruses that infect the human body through the respiratory and digestive tracts. Coxsackievirus A21 (CVA21) is a naturally ICAM-1-targeted RNA virus (Müller et al., 2019). It can target ICAM-1-expressing tumor cells, leading to a systemic antitumor immune response. Phase I of the CVA21 study was conducted as part of an experimental treatment for bladder cancer, and the results confirmed that CVA21 is effective as an antitumor treatment by inducing immunogenic apoptosis in cancer cell lines (Annels et al., 2019).

#### 4.1.8 Vesicular Stomatitis Virus

Vesicular Stomatitis Virus (VSV) is an enveloped, single-stranded negative sense RNA viruses of the family Rhabdoviridae (Nikolic et al., 2018). Two major serotypes of VSV have been identified, with representative strains named Indiana (VSIV) and New Jersey (VSNJV) strains (Velazquez-Salinas et al., 2017). Vesicular stomatitis virus (VSV) can preferentially kill tumor cells with defective antiviral responses and has effective antitumor activity (Durham et al., 2017). Masatsugu Obuchi et al. genetically engineered VSV-hIFNβ that highly expresses the murine IFN-beta gene. While nonlytic against normal human cells, it easily kills tumor cells. Many clinical trials evaluating VSV-hIFNβ for different cancer types, such as head and neck cancer, lung cancer, melanoma, liver cancer, and endometrial cancer, are ongoing (Sterman et al., 2007; Willmon et al., 2009).

#### 4.1.9 Novel Nano-Pseudovirus

Yingzhong Li et al. constructed a novel nano-pseudovirus that mimics the characteristics of oncolytic viruses that trigger immune responses in the body (Li et al., 2020). The nano-pseudoviruses carry self-replicating RNA, which can activate the TLR3 signaling pathway, induce the body’s immune response and promote the immunogenic cell death (ICD) of tumor cells. Preclinical research data show that 60–90% of melanoma and colon cancer lesions can be eliminated by one treatment with nanopseudovirus. The nano-pseudovirus can also effectively inhibit tumors in cases in which lung metastases have already formed. More clinical studies are needed in the future to verify the clinical safety and effectiveness of this approach.

### 4.2 Combined Therapy

The multifunctional characteristics of oncolytic viruses in the process of tumor treatment endow them with great potential for synergy when used in combination with other drugs. At present, substantial evidence indicates that oncolytic virus therapy can induce ICD when used in combination with radiotherapy, chemotherapy and other immunotherapies by enhancing tumor cell antigenicity or susceptibility to immune cells.

#### 4.2.1 Radiotherapy

With the emergence of strategies combining oncolytic virus therapy with radiotherapy, the relationship between the two therapies has attracted the attention of researchers. The combination strategy has shown synergistic antitumor effects in various preclinical studies (Touchefeu et al., 2011; Mell et al., 2017). In some instances, radiotherapy could enhance oncolysis partly because it increases the virus replication rate. Radiotherapy promotes the upregulation of intracellular GADD34 expression, which can functionally replace γ34.5, promote continuous protein synthesis, and increase the virus replication rate (Chou and Roizman, 1994; Kramm et al., 1997). After irradiation, the expression level of the coxsackie-adenovirus receptor (CAR) and/or integrins increases, which leads to an increase in the uptake of the virus (Qian et al., 2005). As a radiation-inducing element, Egr-1 can enhance the expression of transgenes caused by radiation. TRAIL is an apoptosis-related gene (Oikonomou and Pintzas, 2013). Treatment of tumor cells with adenovirus expressing Egr-1/TRAIL combined with radiotherapy can induce high TRAIL expression levels. During this process, the expression of caspase3 and caspase8 can be increased, and their biological effects on apoptosis are also improved. These changes induce tumor regression and improve the survival time of tumor-bearing mice (Zhou et al., 2010; Wang et al., 2012).

#### 4.2.2 Chemotherapy

Chemotherapy is a common cancer treatment. However, tumor cells can develop resistance to chemotherapy drugs, often leading to chemotherapy failure. In recent years, some studies have proven that oncolytic viruses combined with chemotherapeutic drugs have significantly improved efficacy compared with the original single-drug treatment. When ONYX-015 is combined with the chemotherapeutic drugs cisplatin and 5-FU, the objective remission rate can reach 65%, significantly higher than the 15% response rate of ONYX-015 alone (Khuri et al., 2000). Experimental data showed that combining the oncolytic virus H101 with chemotherapy can have antitumor effects by inducing cell cycle arrest in tumor cells (Cun et al., 2012). Combining vincristine (VCR) with oncolytic adenovirus SG600 slowed tumor growth by altering the cell cycle and reducing Akt phosphorylation (which normally induces chemotherapy resistance), thus increasing the sensitivity of the tumor cells to VCR. It was further confirmed that VCR does not affect the replication of SG600, ensuring oncolysis efficacy (Song et al., 2012).

#### 4.2.3 Targeted Therapies

Targeted therapy inhibits the growth of tumor cells by acting on important tumorigenesis and development pathways. The combination of T-VEC and MEK inhibitors enhanced melanoma cell death *in vitro*, suppressed tumor growth and prolonged survival in mouse models (Bommareddy et al., 2018b). Combining si-Notch1 treatment with the oncolytic virus H101 did not affect viral replication and enhanced tumor cell apoptosis compared with the use of the single-agent therapy (Huang et al., 2012a; Yao et al., 2012). H101 combined with si-Bcl acts on the bax-p53 pathway, resulting in cell apoptosis and cell cycle arrest (Zhang et al., 2009; Huang et al., 2012b). GNAQ mutations are known to be associated with uveal melanoma (Jager et al., 2020). Combined therapy using si-GNAQ and H101 inhibits the proliferation of uveal melanoma cells by blocking the phosphorylation of mek1/2 and promoting the phosphorylation of yap (Li et al., 2019).

Immune checkpoint blockade (ICB) aims to resolve tumor escape by reversing tumor immunosuppressive signals. ICB cannot target tumors with low expression levels of immune checkpoint proteins; thus, no antitumor effects are observed in such tumors (Benci et al., 2016). When oncolytic viruses are used in combination with ICB drugs, the viruses can induce a large number of immune cells to infiltrate the tumor (Harrington et al., 2019). The tumor cells will further increase the expression level of checkpoint molecules, such as PD-L1 and CTLA-4, and induce the development of a self-protection mechanism to escape immune attacks. Such combinations expand the scope of ICB drugs and enhance tumor sensitivity to ICB (Raja et al., 2018).

In a murine colon carcinoma model, the combination of a recombinant VV expressing CXCL11 and PD-L1 inhibitors was significantly more effective than monotherapy (Liu et al., 2017). In a phase I clinical study evaluating melanoma patients, combined treatment with T-VEC and the PD1 monoclonal antibody pembrolizumab yielded an objective response rate of 62% among 21 patients, and the complete response rate was 33%. In addition, the expression levels of PD-L1 and IFN-γ were increased, and T cells infiltrated significantly into tumor tissues (Ribas et al., 2017).

#### 4.2.4 Adoptive T Cell Therapy

Adoptive T cell therapy is a powerful strategy to augment T cell immune responses and improve antitumor capabilities (Restifo et al., 2012). Studies have revealed that oncolytic viruses enhance CAR-T cell recruitment by triggering antitumoral immunity (Wing et al., 2018). In a mouse neuroblastoma model, combined treatment with an oncolytic adenovirus (Ad5A24) expressing CCl5 and IL-15 and CAR-T cells targeting the tumor-associated ganglioside gd2 increased the overall survival period and survival rate of mice, and the function of CAR-T cells was strengthened (Nishio and Dotti, 2015). Recently, a new study reported that combined therapy with oncolytic viruses and CAR-T cells can overcome the barriers of the tumor microenvironment and can successfully target and eradicate solid tumors that are otherwise difficult to treat with CAR-T cell therapy alone. In this study, researchers engineered the oncolytic virus to infect tumor cells and force tumor cell surface expression of CD19. Thus, the oncolytic efficacy of CD19-targeted CAR-T cells was enhanced (Park et al., 2020).

## 5. Deficiencies and Improvements for Oncolytic Viruses

### 5.1 Production

The active replication properties of oncolytic viruses pose a unique challenge for monitoring and production. Most viruses multiply in tissues, and methods for high-titer virus production, foreign pathogen detection, viral purity determination and viral replication potential are required (Langfield et al., 2011). Such methods are costly, and existing technologies do not fully meet the requirements, limiting the large-scale production of oncolytic viruses. For some viruses, it is difficult to obtain the extremely high-titer lysate required for clinical doses; thus, oncolytic virus manufacturing is more challenging than the manufacturing of traditional biological products (Ungerechts et al., 2016). In addition, regulatory guidance and quality control systems for the manufacture of oncolytic viruses are not yet complete. It is necessary to consider safety issues related to production and amplification processes.

### 5.2 Barriers

Many factors determine the efficacy of oncolytic viruses in the body. There are some physical barriers in the body, such as macrophages, which can directly capture viruses in organs such as the liver, thereby reducing the viral titer in the body and affecting the oncolytic effect (Fulci et al., 2007; Yang et al., 2015). Macrophages are affected by clodronate; therefore, clodronate treatment may solve this problem (Denton et al., 2018). The use of appropriate cell carriers and liver off-target technology can also reduce the impact of macrophages on the virus (Power and Bell, 2008).

**FIGURE 3:**
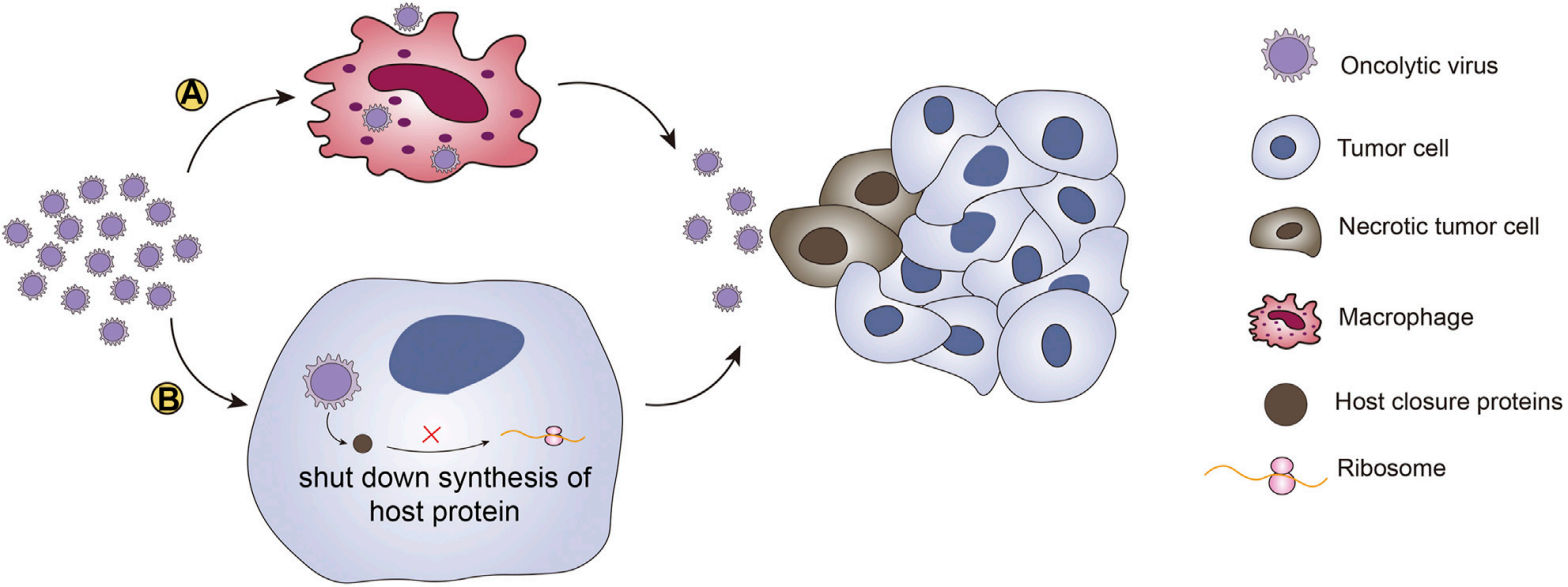
Therapeutic barriers of oncolytic viruses. There are two main barriers to the induction of antitumor effects by oncolytic viruses. **(A)** Macrophages can directly capture viruses in organs such as the liver. Macrophages reduce the virus titer and the antitumor effects through phagocytosis. **(B)** The other barrier for oncolytic viruses is blockade of host protein synthesis. Oncolytic viruses encode host closure proteins that can shut down synthesis of host proteins, destroy pre-existing polysomes, degrade host mRNAs, and interfere with the production of viral offspring. This “host shutoff” is not conducive to virus replication or the expansion of neoantigen-reactive CTLs.

The body’s immune barriers and antiviral responses can inhibit viral replication and lead to resistance to oncolytic viruses. Studies have shown that UL41 of HSV encodes host closure protein, which can shut down the synthesis of host proteins, destroy pre-existing polysomes, degrade host mRNA, and interfere with the production of viral offspring (Su and Zheng, 2017). This blockade of host protein production is not conducive to the expansion of neoantigen-reactive CTLs. To avoid the restriction caused by the blockade of host protein production, it is better to choose viruses that have evolved to inhibit or shut down host cell protein synthesis when selecting oncolytic viruses. Genetic engineering can also be used to insert sequences into the viral genome that regulate biomolecules in the host protein synthesis pathways, thereby avoiding the impact of host protein shutdown on the oncolytic effect. After recognizing PRRs, the virus can promote the production of IFN1 and induce the inherent cellular antiviral response. Another method is the use of histone deacetylase inhibitors (HDIs), which are small molecules that were developed as anticancer agents (Gumbarewicz et al., 2016). HDIs can not only directly kill cancer cells but can also be combined with oncolytic viruses to reduce the antiviral immune response in the body. Nguyen et al. demonstrated that HDIs can enhance the activity of oncolytic viruses in multiple systems *in vivo* and *in vitro* (Nguyên et al., 2008) (Figure 3). However, safety is an important consideration when administering immunosuppressants, and systemic infectious diseases must be avoided.

### 5.3 Safety

The most common adverse events due to oncolytic viruses are low-grade systemic symptoms and local injection site reactions. Fever is the most common treatment-related adverse event, and the viruses can also cause chills, nausea and vomiting, flu-like symptoms, fatigue, and pain (Macedo et al., 2020). A recent clinical study showed that monotherapy and combined therapy with G207 significantly improve the prognosis of pediatric high-grade gliomas, with good safety and tolerability, which indicates that oncolytic viruses are not only safe and effective for adults, but also show good tolerance in children (Friedman et al., 2021). Generally, the safety of oncolytic viruses is acceptable. However, oncolytic viruses are not entirely safe, and some safety problems urgently need to be solved. Although genetically engineered oncolytic viruses have increased targeting and decreased toxicity, they still have off-target and unexpected toxic effects (Buijs et al., 2015). Qiao et al. observed that the use of cyclophosphamide to suppress the immune response and the ablation of neutralizing antibodies can lead to severe reovirus toxicity (Qiao et al., 2008). The oncolytic virus VSV has been shown to have low targeting ability and is mainly found in large numbers in normal tissues. In IFN-α/β knockout mice, VSV can exhibit severe toxicity. Such experiments have proven that although the immune system may reduce the antitumor effect of oncolytic viruses, it also plays an important role in limiting the virus and maintaining safety. Researchers are continuously making efforts to improve tumor targeting and virus potency. To improve tumor targeting ability, sequences of differentially expressed microRNAs are integrated into OVs, which can be adapted to various viruses and reduce damage to healthy cells (Ruiz and Russell, 2015). Ad (CgA-E1A-miR122) was constructed using miR-122 to regulate E1A expression, ensuring selective replication in tumors. Studies have shown that this combination of transcriptional and posttranscriptional regulation allows higher doses of adenovirus for antitumor treatment without liver toxicity (Leja et al., 2010).

## 6. Discussion

Oncolytic viruses are natural or genetically modified viruses that can selectively replicate in tumor cells and have long been recognized for their ability to kill cancer cells. Many years of research have shed light on the mechanism by which oncolytic viruses kill tumors. Oncolytic viruses not only kill tumor cells directly through virus replication but also convert cold tumors into hot tumors by regulating the release of immune-related molecules, enhancing the immunogenicity of tumors, and promoting the maturation, migration and infiltration of immune cells, thereby improving the antitumor immune response. In addition, oncolytic viruses can affect the neovascularization of tumors and inhibit tumor growth. The effectiveness of the oncolytic virus depends on the oncolytic virus infecting tumor cells in a sufficient number. The dose-response relationship of OVs cannot be predicted easily and varies across different methods of delivery (Peng et al., 2006). As the best route of administration is still uncertain, it is essential to explore suitable routes. The most common route of OV administration is intratumoral injection. This approach has advantages in avoiding hemodilution and the anti-vector immunity response. It can also decrease the risk for off-target side effects. However, this method has some deficiencies. Intratumoral administration is associated with a risk for deep tissue injuries and bleeding in some tumors, such as intracranial tumors and malignancies near the edge of the liver. Meanwhile, the lack of secondary viremia may result in limited immune activity (Andtbacka et al., 2016). The advantage of intravenous delivery is that multiple sites (including metastasis sites) can be targeted regardless of tumor location; additionally, the therapy is easy to administer (Bommareddy et al., 2018a). However, hemodilution is a limiting factor (Breitbach et al., 2011). The tumor is not accessible when it comes to isolation in non-target organs or the inability of the virus to extravasate through the vasculature (Ferguson et al., 2012). There are other routes of administration for specific types of tumor, such as intraperitoneal delivery for digestive tract malignancy (Kulu et al., 2009), isolated limb perfusion for extremity sarcoma (Pencavel et al., 2015) and aerosol delivery for lung tumors (Hong et al., 2015). Developing suitable drug delivery systems is an important direction for oncolytic virus research. Recently, many clinical trials evaluating oncolytic viruses have been carried out, involving different types of viruses and tumors. Treatment methods also vary from single-drug therapies to multidrug combination therapies. Most trials have achieved good results. Although oncolytic viruses have immeasurable application potential and can be marketed as therapeutic agents, some barriers exist to their production and application. Current molecular biotechnology strategies have enhanced the targeting and killing effects of oncolytic viruses; however, to develop tumor treatment methods with higher curative effects and lower adverse reaction rates, further development is needed. Additionally, because the production technology used to generate oncolytic viruses is not perfect, and there is no unified standard for the supervision and quality inspection of industrial production, there are still barriers to the large-scale production and application of oncolytic viruses. Therefore, more extensive research on oncolytic viruses is needed in the future to develop more optimized treatments.

## Glossary

5-FU: 5-fluorouracil
Ad-199T: Adenovirus-199T
BBB: Blood-brain barrier
CAR: coxsackie-adenovirus receptor
CAR-T: Chimeric Antigen Receptor T-Cell Immunotherapy
cGAS: cyclic GMP–AMP synthase
CTL: cytotoxic T lymphocyte
DAMP: damage-associated molecular pattern
DC: dendritic cell
DC-Mel: dendritic cells loaded with human melanoma Mel888 cells
DCN: decorin
dsDNA: double-stranded DNA
dsRNA: double-stranded RNA
ECM: extracellular matrix
FDA: Food and Drug Administration
FdUMP: fluorodeoxyuracil monophosphate
FUTP: fluorouracil triphosphate
HCC: hepatocellular carcinoma
hTNF-α: human tumor necrosis factor α
HSP: Heat shock protein
HSV: herpes simplex virus
ICB: Immune checkpoint blockade
ICD: immunogenic cell death
mDC: mature dendritic cell
MV: measles virus
NK: natural killer cell
NDV: newcastle disease virus
PAMP: pathogen-associated molecular pattern
Pexa-Vec(JX-594): pexastimogene devacirepvec
ssDNA: single-stranded DNA
ssRNA: single-stranded RNA
STING: stimulator of interferon genes
TAA: tumor-associated antigen
TIL: tumor infiltrating lymphocyte
UL41: HSV virion host shutoff protein
VAP: virus adsorption protein
VCR: vincristine
VDEPT: virus-directed enzymatic prodrug therapy
VEGF: vascular endothelial growth factor
VSV: vesicular stomatitis virus
VV: vaccinia virus
